# Between worlds: Cis-and trans-identifying diaspora Middle Eastern and North African (MENA) women in Ontario, Canada on the intersections of gender, sexuality and sexual health

**DOI:** 10.1371/journal.pgph.0003854

**Published:** 2024-11-08

**Authors:** Roula Kteily-Hawa, Olesya Falenchuk, Bessma Momani, Vijaya Chikermane, Susan Bartels, Praney Anand, Rania Younes, Nahla Abdel-Tawab, Nadia N. Abuelezam, Lina Hammad, Tina Pahlevan, Rama Eloulabi, Ahmad Ezzeddine, Anmar Al-Ezzawi, Mohammad Akel, Mona Loutfy

**Affiliations:** 1 Family Studies and Human Development, Faculty of Health Sciences, Western University, London, Ontario, Canada; 2 Ontario Institute for Studies in Education, University of Toronto, Toronto, Ontario, Canada; 3 Department of Political Science, University of Waterloo, Waterloo, Ontario, Canada; 4 7.10 Stories, Toronto, Ontario, Canada; 5 Department of Public Health Sciences, Queen’s University, Kingston, Ontario, Canada; 6 Alliance for South Asian AIDS Prevention, Toronto, Ontario, Canada; 7 Ontario College of Art and Design University, Toronto, Ontario, Canada; 8 Population Council, Cairo, Egypt; 9 College of Human Medicine, Michigan State University, East Lansing, Michigan, United States of America; 10 YSMENA Study, Canada; 11 Dalla Lana School of Public Health, University of Toronto, Toronto, Ontario, Canada; 12 Department of Psychology, Western University, London, Ontario, Canada; 13 HIV & AIDS Legal Clinic Ontario, Toronto, Ontario, Canada; 14 Women’s College Hospital, University of Toronto, Toronto, Ontario, Canada; University of Toronto Temerty Faculty of Medicine, CANADA

## Abstract

Youth Sexual Health and HIV/STI Prevention in Middle Eastern and North African Communities (YSMENA) is the first community-based research study in Canada to explore key determinants of sexual health among diaspora Middle Eastern and North African (MENA) women living in Canada. Our objectives were to identify the factors influencing sexual health for MENA youth and grow an evidence base to strengthen the sexual health response for MENA communities. Using mixed- method design, data were gathered through a quantitative socio-demographic survey and qualitative focus groups with 24 women-identifying MENA youth (16–29 years) living in Ontario, Canada. Six (6) focus groups were held virtually via Zoom, with heterosexual, lesbian, bisexual and queer (LBQ), and trans women. Members of each group participated in two sessions as per the sequential critical dialogical method and transcripts were coded in NVIVO. While participants identified with diverse sexual orientations and across the trans-feminine gender spectrum, key commonalities surfaced, namely the pressures to conform to familial expectations and the barriers faced when accessing healthcare. The trans group experienced compounded levels of exclusion given their unique positioning. Although the challenges faced by the group were deeply entrenched in patriarchy, heteronormativity and internalized shame, many participants demonstrated resilience and self-acceptance that enabled them to forge pathways to health. Findings have important and valuable implications for community-based health programs targeting MENA women as well as healthcare practitioners and service providers.

## Introduction

In recent decades, gender as a social construct has gained deserved traction as a well-documented determinant of health and social outcomes [1]. Women’s health outcomes, particularly in sexual health, are deeply influenced by the sexist and gendered practices they face in society [2, 3]. Gendered experiences in health are further complicated by intersecting determinants such as class, age, or racialization [2, 4]. In Canada, health researchers highlighted the importance of research involving gender relations alongside culturally accessible health services [1, 5]. This paper is situated at these intersections. It examines the lived experiences and sexual health needs of Middle Eastern and North African (MENA) diaspora youth identifying along the transfeminine gender spectrum.

The sexual health outcomes and practices of young women from MENA countries represent an under researched area [6]. While particular women’s challenges in the region have drawn attention and gained momentum(e.g., rights to education, political participation, or work), issues of sexual and reproductive rights are more sensitive and have only recently gained traction [6]. The dearth of literature on sex and sexual health behaviors of MENA women warrants attention as the MENA region experiences increased human immunodeficiency virus (HIV) infections while facing unique challenges in navigating care for HIV and sexually transmitted infections (STIs) [7].

Between 2001 and 2016, MENA has experienced a 31% increase in HIV infections, despite global rates of new HIV infections dropping in that period [8, 9]. In the context of gender and HIV, gender inequality is considered a key factor contributing to increased rates of infection as women often have less access to information, reduced power to negotiate safer sex, and limited mechanisms to cope with the stresses of infection [10].

Although gender inequality is an established driver of HIV infection, a scoping review of research on HIV, STIs and sexual practices among MENA youth demonstrated limited literature with gender as a focus. Existing research focuses on people who use drugs, people who identify as gay and/or men who have sex with men (MSM), and/or people in prison. Select studies highlighted risks as experienced by female sex workers [11], trans women [12] and/or female university students [13, 14]. Few take a sociological approach to sexual health grounded in intersectionality or gender, and none have considered young diaspora MENA women or diverse sexual and gender identities.

In Canada, the highest proportion (62%) of newcomers come from Asia (including the Middle East), with many making their home in the province of Ontario [15]. After arrival to Canada, many face unemployment, language barriers, structural discrimination, limited social support systems, and cultural barriers that can increase their vulnerability to diminished sexual health outcomes, STIs and HIV [16]. Despite a strong body of evidence around their sexual health issues while living in the MENA region, little is known of the factors that influence newcomers’ sexual health or access to health services after they migrate to Canada. Studies situated in the sexual health needs of diaspora MENA women in Canada and North America are also limited [17–19], especially ones inclusive of young women who identify across the transfeminine gender spectrum [20].

Knowledge in this area can fill important gaps on how to tailor and craft gender-based, culturally relevant health responses for the growing MENA community as newcomers re-settle into Canadian society and healthcare systems. To that end, the YSMENA study (Youth Sexual Health and HIV/STI Prevention in Middle Eastern and North African Communities) was initiated to address the dearth of literature on the sexual health realities of diverse diaspora MENA youth in Canada’s most populous and largest immigrant-receiving province—Ontario. It is the first community-based research study in Canada to (a) center the voices of MENA youth who identify across the spectrum of sexual and gender identity and (b) utilize the theoretical frameworks of intersectional feminism and queer theory.

Herein, we present mixed methods results and findings from the women-identifying participants of the larger YSMENA study with clear implications for health and social care work.

The term *woman* was used to refer to all female-identifying participants. They included (a) *straight* (attracted to the opposite sex); (b) *cisgender* (people who identify as a woman–both their sex assigned at birth [female] and gender); (c) *queer* (sexual and gender identities other than straight and cisgender); and (d) *trans* women (assigned male at birth but identify as a woman).

### Theoretical frameworks

Informed by critical queer theory, furthered by Butler and Harris, and the feminisms of racialized women theory, furthered by Lorde and Anzaldua, the YSMENA study challenges the traditional segregation of women across sexual and gender binaries to explore a more nuanced lens [21–24]. More recently, MENA researchers and activists have highlighted the importance of conversations of sexual health and *women* being inclusive of trans women, a community often overlooked in dialogue of gender and sexual health [12]. *Women* were termed as those who self-identified as such, and our thematic findings share the diverse ways in which they experienced aspects of sexual health within and outside of their unique identities.

Our research was also supported by the theoretical underpinning of intersectionality, building on the understanding that there are multiple overlapping systems of privilege and oppression that intersect in determining the outcomes of one’s life [25]. This is reflected in the areas of inquiry that explore individual and structural factors such as cultural identity, gender, family, religion, race, school, and access to sexual healthcare. Intersectionality is rooted in a racialized feminist lens that originally explored interlocking systems of race, class, gender, sexual orientation and other determinants that define women’s lives [26]. It is especially helpful in the analysis of gender and health as related to the women-identifying participants in our study. Our discussion points also situate findings and results within the lens of queer theory and intersectional feminism to further our understanding of the data.

Regarding terminology, MENA refers to countries in the Middle East and North Africa, that share certain commonalities in socio-political history, language, religion and/or culture [27]. The label *Middle East* is a colonial term forced on the MENA region during European occupation [28, 29]. Alternative terms include *West Asian* or *Southwest Asian* and *North African* (SWANA). That said, we used MENA as a community and regional descriptor because it aligns with United Nation’s (UN) current terminology. Statistics Canada uses the terms *West Asian* and *Arab* to indicate two separate groups.

### Research objectives

The study’s central objectives were to (a) engage in developmental research that builds community and youth capacity in knowledge generation and sexual health; (b) understand how the social environments and relationships of cis and trans diaspora MENA women, in the contexts of their homes, schools, and communities, shape their identities and influence their sexual health; and (c) bridge existing gaps in the literature relevant to the MENA community and amass evidence-based research to inform future health programming and policy.

## Methods

### Ethics statement

The study received ethics approval from the University of Toronto’s Research Ethics Board (# 38931) and Brescia University College’s Research Ethics Board. Formal consent was obtained from all participants in writing through an informed consent form administered via REDCap.

### Community-based participatory approach

The YSMENA study was initiated in a community-based health setting by the *Alliance for South Asian AIDS Prevention* (ASAAP) and long-time community researcher and supporter, study principal investigator and first author RKH. ASAAP is an AIDS Service Organization based in Canada’s most populous city, Toronto, Ontario. ASAAP offers HIV and STI prevention and support services for South and West Asian diaspora. The YSMENA study was a research response to the growing number of MENA community members accessing ASAAP’s sexual health needs of newcomer and lesbian, gay, bisexual, transgender, and queer (LGBTQ+) MENA community members.

The YSMENA study was grounded in community-based participatory research (CBPR) principles that prioritized placing MENA youth and community members as drivers of the study. CBPR centers the voices of participants and community in leading research and equalizes power between researchers and community members to support reflective action and knowledge gains through the research process [30–32]. YSMENA’s mixed-method study protocol incorporated both quantitative and qualitative data collection purposefully designed and implemented by MENA-identified community members and peers.

In keeping with CBPR, a peer leader model was adopted with the objective of building community capacity in research and knowledge generation. To that end, six (6) MENA Youth *Peer Research Associates* (PRAs) were recruited. All PRAs underwent a two-day extensive training in community-based research principles: community and participatory research, social determinants of health, anti-oppression frameworks, intersectionality, research ethics and confidentiality, informed consent, survey administration, managing difficult conversations, focus group facilitation, how to unpack privilege, and more.

In addition, all PRAs were required to complete two (2) certificate-based training courses with the *Canadian AIDS Treatment Information Exchange* (CATIE) and the Tri-Council Policy Statement: Ethical Conduct for Research Involving Humans (TCPS 2) offered through the Government of Canada’s Panel on Research Ethics. This extensive training equipped PRAs to support the development of research tools and materials, participant recruitment and promotion, and data collection with them facilitating qualitative focus groups, and administering quantitative socio-demographic surveys.

### Participant recruitment and sample frame

Discussion amongst ASAAP, the PRAs, and other community partners identified a need to recruit youth that represent sexual and gender identity spectrum. Consistent with the first author’s [33] MENA youth scoping review, the study also identified key youth populations who experience disproportionate rates of HIV and STIs: (a) gay and bisexual men and men who have sex with men (gbMSM); (b) trans-identifying women; and (c) people, who through close proximity to high-risk groups, are at the risk of contracting HIV such as university students. Furthermore, (d) youth aged 16–29 who identified as being of first or second-generation MENA ethnic background were recruited into one of five (5) sub-groups: heterosexual cis women, heterosexual cis men, lesbian, bisexual and queer (LBQ) cis women, gbMSM, and trans women. Overall, N = 56 youth were recruited. Nearly half (n = 24, 42%) identified as women and are the focus of this paper: 14 as cis heterosexual women, seven as trans women, and three as cis LBQ women.

Participant recruitment occurred between February 1^st^, 2021 and March 12, 2021 during COVID-19 using study flyers, word of mouth, the PRAs, list servs of partner organizations, and private social media groups, such as *LGBTQ+ Middle Easterners and North Africans in Canada*. Participants were recruited from across Ontario (Ottawa, Greater Toronto Area, Hamilton, Kitchener, Cambridge, Waterloo, London, and Windsor).

### Instrument development

The research team developed a three-part quantitative survey instrument. Section 1 consisted of 27 closed-item demographic and socio-economic questions. Section 2 comprised 10 closed-items questions about sexuality and sexual health. A community advisory board provided feedback on the different sections to make sure it was culturally acceptable and relevant. Lastly, the instrument was pilot tested with MENA peers and then improved and finalized.

In section 3, self-identifying heterosexual men, heterosexual women, lesbian, bi and queer women were asked to complete the previously validated *Denver SHIV Risk Scale* [34]. Self-identifying MSM and transgender women were asked to complete the previously validated *HIV Incidence Risk Index* (HIRI) [35].

The research team also developed a qualitative journaling survey containing questions (available from the authors) about participants’ (a) experiences with health care access (seven questions) and (b) the intersection of sexual and ethnic identities (five questions). These 12 questions were similar but tailored for each self-identifying sub-group (e.g., LBQ women, and transgender women).

The focus group interview guide comprised two sections with five questions each along with pre-identified probes (available from the authors). Participants were advised that the questions were applicable to them in particular or MENA youth in their community: As with the individual journal, questions were similar yet tailored for the specific sub-group and pertained to (a) the intersection of sexual and ethnic identities and (b) HIV-related risk and protective factors.

### Data collection

Data collection took place between February 15^th^ and June 4^th^ 2021 and unfolded in three (3) phases. Phase one asked participants to sign informed consent forms before completing the socio-demographic and HIV risk survey using the REDCap application (approximately 30 minutes). Survey responses were anonymous. In Phase two, after completing the quantitative survey, and still in REDCap, participants were directed to complete a qualitative individual journal (approximately 30 minutes).

In Phase three, additional qualitative data were collected through sequential dialogical focus groups, which required engaging the same participants over separate sessions for a deeper discussion and dialogue. This method offers deeper engagement and reflection than a single-session focus group, and it is especially applicable to sensitive health subjects and/or vulnerable populations [36]. This three-phased approach aligned with CBPR principles and ensured that participants could share their thoughts and experiences using the method they felt most comfortable with–either through writing (individual journals) or oral sharing (focus groups). Also, each focus group was hosted by three (3) of our own especially trained PRAs, with assigned roles of either lead facilitator, co-facilitator, or field note-taker.

**Fig 1:**
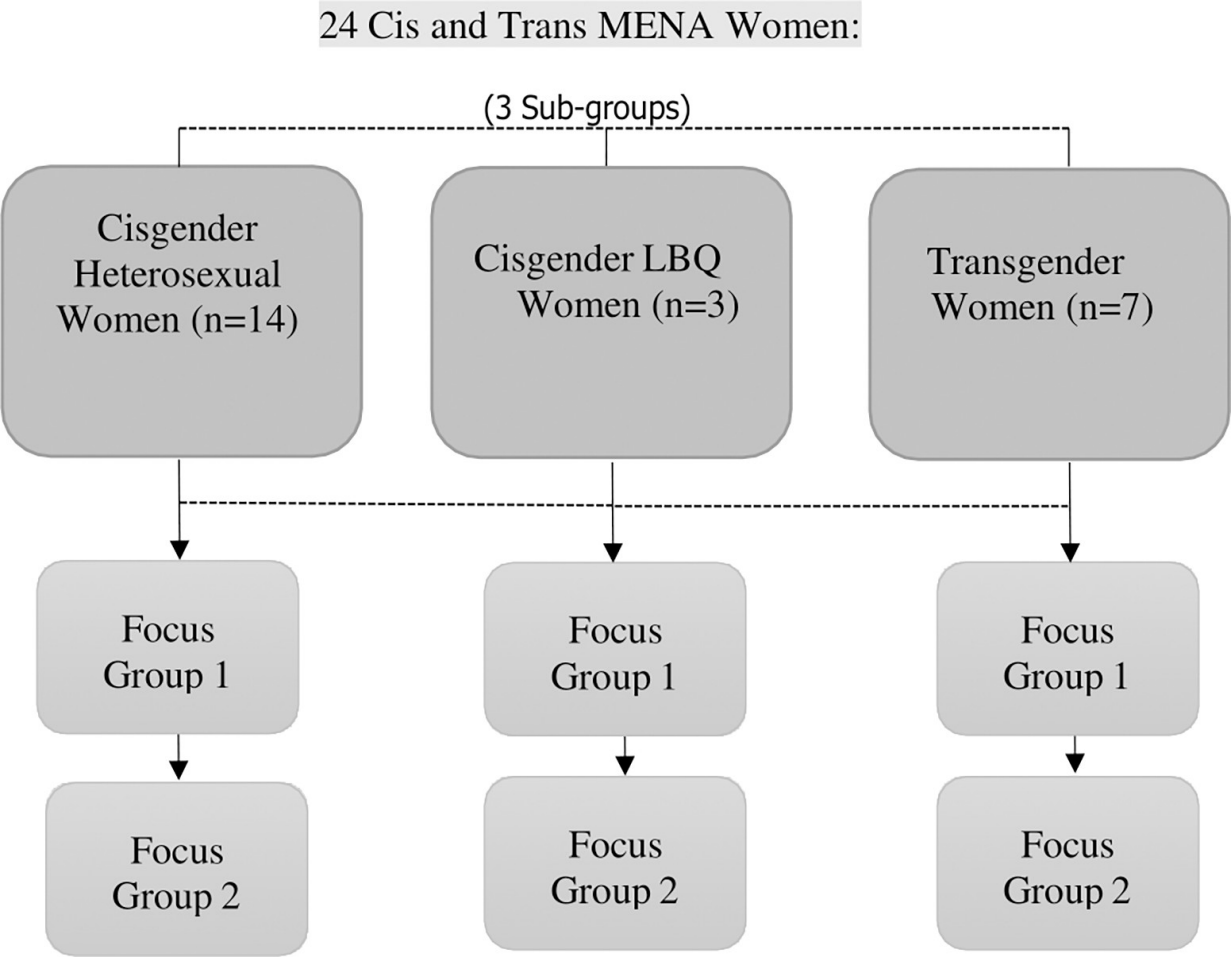
Sequential dialogical focus group design.

In keeping with the sequential dialogical focus group method [36], two focus groups were held with the same participants recruited for each sub-group for a total of 14 focus groups and 56 youth participants. For the study reported herein, a total of six (6) focus groups were held with 24 women-identifying youth participants under three (3) sub-groups, cis heterosexual women, cis LBQ women, and trans women (see Fig 1). The trans women’s focus groups were conducted in Arabic given the language barriers experienced by the group’s participants who were primarily newcomers. This transcript was then translated to English for analysis. All focus group data were collected online via Zoom and lasted on average 2–21/2 hours.

### Data analysis

Quantitative data were analyzed using descriptive statistics (frequency counts, percentages, and Standard Deviation [SD]). This statistical protocol served the purpose of the quantitative strand corroborating qualitative findings. Statistical results served a supportive role not a substantive or central role.

All data were coded in NVivo and then underwent a thematic analysis: the focus group transcripts, the PRAs’ field notes completed following each focus group session, and the participants’ journals. Several sources informed the thematic protocol [37–40]. Succinctly, analysis entailed iterative inputs and review from the research team and PRAs. Coding nodes were determined through discussion with the research team and PRAs who then reviewed preliminarily results through two (2) structured review sessions to triangulate and strengthen the findings. This interactive and iterative process has offered a rich and nuanced set of findings.

Owen’s (40) criteria for when a theme exists were applied: (a) the same thread of meaning is found even when different words are used *(recurrence);* (b) key words, phrases, or sentences are repeated throughout the data set (*repetition*); or (c) the incidence of comments may be small, but the passion and emotions are high (*forcefulness*). Krueger [41] Morgan’s [42] criteria were also used; (d) the *frequency* or how often a topic is discussed and (e) the *extensiveness* of the comments (i.e., the number of people who mentioned the idea). Owen strongly asserted that a theme need not meet all criteria [40].

Herein, respectful of word count and length of paper, single quotes *exemplifying* a finding were often chosen rather than multiple quotes from several people, which are available from the authors. Regarding study rigor, data from the small sample frame were deemed *authentic* (real for the participants), *confirmable* (researchers were neutral when interpreting data) and *credible* (obtained a faithful and accurate representation of their reality) [43]. Meeting these criteria lends support to our conclusions and recommendations.

## Results and findings

Results and findings from this mixed methods study pertained to the women-identifying groups whose experiences of navigating sex, sexuality, and sexual health were largely determined by their gender identity. Twenty-four (24) women-identifying participants completed the survey. They averaged 26 years in age (SD = 3.92), ranging from 21 to 38 years. Most (*n* = 17, 70%) were born in a country other than Canada and reported Middle Eastern ethnicity (*n* = 19, 78.3%). More than half (*n* = 14) had lived in Canada less than nine years. Most (*n* = 18) had completed undergraduate university education or higher. About half (*n* = 13) reported Islam as a religious affiliation. In terms of financial support, one third (*n* = 8) had received social or disability assistance. About one third reported problems with their current living space–virtually all the trans women (see Table 1).

**Table 1:** YSMENA study: Demographic characteristics of the women-identifying participants.

	Hetero Cis (n = 14)	LBQ Cis (n = 3)	Trans (n = 7)	All women (N = 24)
**Age, mean(SD)**	24 (2.11)	25 (2.08)	27 (7.59)	26 (3.92)
**Age, range**	21–28	23–27	22–38	21–38
**Place of birth, n (%)**				
**Canada**	5 (33.3)	1 (33.3)	1 (16.7)	7 (30.0)
**Other**	9 (66.7)	66.7 (2)	6 (83.3)	17 (70.0)
**Time living in Canada, n (%)**				
**Entire/most of life**	7 (50.0)	1 (33.3)	1 (12.5)	9 (34.8)
**10+ years**	2 (16.7)			2 (8.7)
**4–9 years**	4 (25.0)	1 (33.3)	1 (12.5)	6 (21.7)
**1–3 years**	1 (8.3)	1 (33.3)	4 (50.0)	6 (26.1)
**Less than 1 year**				
**Prefer not to answer**			2 (25.0)	2 (8.7)
**Ethnicity, n (%)**				
**Middle Eastern**	11 (75.0)	3 (100.0)	5 (75.0)	19 (78.3)
**North African or Multiethnic**	3 (25.0)		2 (25.0)	5 (21.7)
**Status in Canada, n (%)**				
**Canadian citizen**	9 (66.7)	2 (66.7)	1 (12.5)	12 (47.8)
**Permanent resident**	4 (25.0)	1 (33.3)	4 (62.5)	9 (39.1)
**Other**	1 (8.3)		2 (25.0)	3 (12.9)
**Level of education, n (%)**				
**High school or less**	1 (8.3)		5 (71.4)	6 (27.3)
**Undergraduate university**	10 (75.0)	2 (66.7)	2 (28.6)	14 (59.1)
**Languages spoken at home, n (%)**				
**English + other**	11 (83.3)	2 (75.0)	3 (50.0)	16 (65.5)
**Only English**	3 (16.7)	1 (25.0)	4 (50.0)	8 (34.5)
**Language spoken with friends, n (%)**				
**English + other**	10 (66.7)	3 (100.0)	5 (75.0)	18 (77.0)
**Only English**	4 (33.3)		2 (25.0)	6 (23.0)
**Living with chronic condition, n (%)**	2 (16.7)	1 (33.3)	1 (12.5)	4 (17.4)
**Sexual orientation, n (%)**				
**Heterosexual**	14 (100.0)		2 (25.0)	16 (60.9)
**Lesbian**		1 (33.3)	2 (25.0)	3 (13.0)
**Gay**			2 (25.0)	
**Bisexual**		1 (33.3)		3 (13.0)
**Pansexual**			1 (12.5)	1 (4.3)
**Other**		1 (33.3)		1 (4.3)
**Prefer not to answer**			1 (12.5)	1 (4.3)
**Islam as religious affiliation, n (%)**	6 (41.7)	2 (66.7)	4 (62.5)	12 (52.2)
**Employed before COVID-19 pandemic, n (%)**	12 (83.3)	2 (66.7)	2 (37.5)	16 (65.2)
**Received social or disability assistance, n (%)**	1 (8.3)	1 (3.3)	6 (75.0)	8 (34.8)
**Sources of income, n (%)**				
**Loans**		2 (50.0)	4 (66.7)	6 (25.0)
**Used personal savings during COVID-19 pandemic**	10 (71.4)			10 (41.7)
**Used other person’s income during COVID-19 pandemic**	4 (28.6)	2 (50.0)	2 (33.3)	8 (33.3)
**Income, n (%)**				
**>$50,000**	6 (45.5)	2 (50.0)		8 (33.3)
**<$10,000**	4 (27.3)	2 (50.0)	1 (20.0)	7 (27.8)
**Living arrangements, n (%)**				
**Own condo/house**	2 (16.7)			2 (9.5)
**Rented apartment/condo**	6 (41.7)	1 (33.3)		7 (28.6)
**Lives with roommate/friend**			7 (100.0)	7 (28.6)
**Lives with parents**	5 (33.3)	2 (66.7)		7 (28.6)
**Other**	1 (8.3)			1 (4.8)
**Current living situation is safe, n (%)**	14 (100.0)	3 (100.0)	3 (50.0)	20 (85.7)
**Problems with current living space, n (%)**	2 (16.7)	0.0	5 (83.3)	7 (29.2)
**Relationship status, n (%)**				
**Single**	6 (41.7)	2 (66.7)	4 (62.5)	12 (52.2)
**In a relationship, living together**		1 (33.3)		1 (4.3)
**In relationship, but not living together**	6 (41.7)		1 (25.0)	7 (30.4)
**Legally married**	1 (8.3)			1 (4.3)
**Common-law relationship**	1 (8.3)			1 (4.3)
**Had consensual sex in last 6 months, n (%)**	12 (83.3)	2 (66.7)	2 (28.6)	16 (63.6)

**Fig 2:**
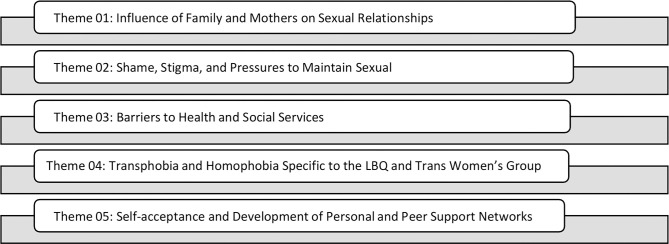
YSMENA study themes.

Five themes (see Fig 2) were identified: (1) influence of family and mothers on sexual relationships; (2) stigma, shame, and pressure to maintain sexual innocence; (3) barriers to health and social services; (4) homophobia and transphobia; and (5) self-acceptance and the development of peer support networks. Each theme (a) includes elements that were either common and/or specific to the female sub-populations and (b) recognizes the factors that made participants’ experiences unique from one another. As a caveat, Owen’s forcefulness criterion played a key role when identifying themes generated from small sample frames [40].

### Theme 01: Influence of family and mothers on sexual relationships

Participants across all the women-identifying groups discussed the influence that their family had or has on shaping their attitudes and behaviors towards sexual relationships. Common references were made to the stigma surrounding sex and sexuality that minimized the possibility of open conversation with parents and/or other family members. The stigma around sex learned through family attitudes and social norms often manifested as shame around women’s sexual relationships and activities. Many talked about needing to unlearn this stigma to have fulfilling relationships that were not based on fear. This comment exemplifies this finding: *“There’s internalized shame in terms of the way I was raised*, *and things I was taught to believe*, *and then the things that I’m doing*, *even though I know are natural and normal*, *there’s still a lot of guilt involved*.*”*

While most participants spoke of how uncomfortable they would be talking about their sexual relationships with their families, many also expressed a desire for that comfort with their mothers. As one participant remarked:

*Sometimes I feel uncomfortable around certain family members, but definitely just the fact that even my mom, and I’m very lucky with the mother, but I still don’t feel comfortable with those conversations and when I was 15 or 16, looking back I wish I was able to go up to her and say* ‘*you know mom if I do meet a guy that I like and if I’m in a very comfortable and in a respected relationship, you know what steps do I have to take to practice safe sex’.*

Even when participants shared a good relationship with their mothers, talking with them about sex was not an option. One participant stated, *“I know myself*, *like I’m not going to go to my mom*. *My mom’s a gynecologist*, *and I never go to my mom for sexual health anything which is*,*[long pause] it’s so sad*.*”* When asked about what they would like from these relationships, participants discussed the value of knowing there is a comfortable and non-judgmental space at home to have those conversations. Although they might choose to go elsewhere, they would still like to know that they could talk to their family, especially their mother.

Cis LBQ-identifying women struggled to find balance between their family, cultural communities, and their queer identities. They faced compounded stigma as queer and sexually active women, which manifested in their relationships either by keeping their sexual lives hidden or distancing from their families. One participant lamented *“I have a very rough relationship with them [family]*, *I feel as though I resent them a lot because I know they will never truly accept me”*

Trans women placed critical importance on the role of their families, especially their mothers, in supporting them through navigating the challenges of asserting their gender identity. More than the other sub-groups, trans women discussed the relationship with their mothers in the context of familial bonds. Culturally, the identity or role of motherhood is a powerful one that holds tremendous emotional weight. This was evident in the way participants discussed these relationships. For example,

*I consider a mother to be a great force. You should try to please your mother and not rebel against her, because she is the only person who will be by your side*. *Even your father can change or stand against you in society, but your mother will stand by your side no matter the circumstances. So my mother protects me from everything.*

Although some participants talked about openness with their mothers, primarily in relation to their gender identity rather than sexual practices, many went to great lengths to hide both from their families. Some participants who felt they could not be open about their identity, talked about having to sever relationships with their family and the MENA community. One participant poignantly said, *“I’m not part of the community*. *I don’t know anybody in the Arabic community*. *I don’t speak to my family*.*”*

All groups talked about the intentional choices they made in hiding aspects of their lives from family members in order to keep peace at home and/or to maintain their familial relations. Many women across all sub-groups seemed to place a greater value on maintaining their family connections than being open about their sexual lives or gender identity. This is exemplified in the following comment:

*We (my family) have a great relationship together so I would never do anything to upset them. There are restrictions, if you step past these, the other person will get upset at you and because you love them so much, you can’t give them up or remove them from y*our life*. My mother in particular remains a barrier stopping from these things or makes me reconsider. There is a struggle inside me so that I do not lose this person.*

Many participants believed that hiding their sexuality was a necessary choice for the good of their families. The stress of juggling these aspects of themselves was seen as worth it if it meant maintaining a conflict-free relationship with family members. One participant commented that “*I do respect my family and I’m very family oriented*, *so I wouldn’t do anything to make them lose respect for me such as openly date a woman*, *or talk about any sexual interactions even with males*.*”*

Some participants talked about coming to an understanding with their parents or families where they just had to accept them for who they were without expecting them to change. In a powerful example, one participant said, *“I spent an extended period of time with my parents and it was just a confirmation for me of how different we are*. *They were like ‘we’ll just never be able to understand you and that’s okay*, *and you’ll never understand us’ and we kind of got to the conclusion that we’re different and that’s okay*.*”*

### Theme 02: Shame, stigma and pressures to maintain sexual innocence

Women across all groups referenced the cultural pressure they experienced to maintain *sexual innocence* in accordance with the idea of staying “pure” or being a virgin for marriage. Stemming from deep-seated patriarchy, the notions of sexual innocence applied to these women. The pressure to be virgins gave way to considerable shame in how they approached sexual activity. Cisgender women pointedly discussed the double standards applied where they may have been ostracized for sexual behavior, whereas their male counterparts would not be affected. As one participant observed, *“I think that is very prevalent in the community where the parents and grandparents are always much harder on the girls than they are on the guys”*.

Cis women (heterosexual and LBQ) used the term “purity culture” to describe the gendered expectations on them to remain “pure and clean” as sex would sully them or make them undesirable. This “dirtiness” associated with sex also seemed to greatly impact and influence their sexual choices. This participant’s comment exemplifies this finding:

*I was raised into "purity culture" and for a long time worried if I had sex*, *if I somehow impacted my hymen, it could prevent me from being with the love of my life. That he might reject me, that men like to "play around" but at the end of the day want to be with a virgin. I had to unlearn a lot of my religious and cultural conditioning before I had penetrative sex for the first time. It created a lot of unnecessary anxiety and fear as I was exploring sexual activity and my own boundaries.*

In some cases the pressure to maintain virginity led to risky sexual activity. One participant declared that *“I know people who before participating in any sexual activity are like*, *‘as long as I don’t lose the hymen and as long as I’m not shamed in society’ it’s okay*, *so they actually just have anal sex*.*”* In other instances the shame women felt led to troubling compromises in terms of negotiating healthy relationships as illustrated in this comment. *“I was with a guy that I continued being with*, *despite the fact that it was an abusive relationship almost close to physically abusive*, *but we didn’t get there*, *quite yet*. *But only to prove that I’m going to end up*

*with him to prove that I’m not a whore to prove that I am doing all of this with a guy that I love that I’m going to end up with*.*”* The shame some women were made to feel for being sexually active was tied to potentially dangerous health and safety outcomes.

Sex stigma created real barriers for young women to access health resources and obtain useful information related to sex and sexuality. These pressures not only affected their decisions related to sexual activity or relationships but also influenced their access to information or services. One participant lamented that she *“can’t even walk into a pharmacy to ask for a pregnancy test*, *there’s so much fear and there’s so much shame*, *so we don’t talk about it with each other*, *but we do it [sexual practices]*, *you know*, *because we want to form fulfilling meaningful relationships*.*”* The stigma that led to participants being cut-off from resources also gave way to misinformation as women then heavily relied on unverified online sources or friends for sexual health information. As one participant explained,

*there is a lot of misinformation, taboo, judgement, and prejudice behind sexual practices/life. I have not had any education about sexual activities up until, well even till today*. *Even around my period, I never had a conversation with either parent about it. This built a prejudice in my mind that it’s all associated with shame, taboo, and disgust. This shaped how I viewed myself and my sexual needs from a young age. I am currently working on correcting that as best as I can but it ’s hard.*

Reliance on the Internet and friends for information is also supported by our quantitative survey results, which indicated that over 80% (*n* = 19) of women-identifying participants listed ‘the Internet’ or ‘friends’ as their main source of sexual health information. Some participants expressed concern over the impacts that shame has on women’s ability to equip themselves with the right information and make informed decisions about their sexual lives. One participant elaborated, *“It makes me sad when I see like a lot of young girls*, *in their mid to late teens*, *they don’t have that information and they’re guilty and shamed into*, *you know*, *not learning about it*.

*But I think it’s extremely dangerous, because, like I said, they’re going to go through with it nine out of 10 times anyways, so you might as well educate them and make sure they’re taking the right precautions.*

When expressing shame, participants in the trans women’s group were clear to denounce sexual activity. The stigma they felt largely reflected community members’ assumptions about sex work. *“So when it comes to the Arab community which is closed off*, *in terms of religion and tradition*, *if they see me*, *because it is something that is apparent*, *they do not know who I am*, they do not know who ** is, or who ** is. *They will see prostitution*.*”* Participants wish to be distanced from assumptions of sex work also led to distance from peers in the larger trans community. One participant admitted that *“I stopped meeting trans people like me here*. *Why*? *Ask me why*. *Because when I hang out with them*, *there is no conversation that you can have as friends or as a family*. *The conversation consists of sex and what kind of client she got and how much the client paid her*.”

In distancing from the community, some of participants adopted d stigmatizing and/or judgmental language related to sex work or other taboo topics such as substance use. As one participant opined, “*Sex work and drugs and drinking as in alcohol and such*, *which also lead to these [sexual] actions*. *There are also things that I consider unnatural…I mean*, *sorry*, *I am speaking my personal opinion*.” For some participants who spoke of sex this way, the stigma manifested as internalized shame. Regardless of their comments about not being sexually active, survey results affirmed that nearly two-thirds (*n* = 14, 58%) of trans women had engaged in consensual sex over the previous six (6) months. Additionally, participants raised conversations about STI testing and taking Pre-Exposure Prophylaxis (PrEP) to prevent getting HIV, further suggesting that their vocal disdain (i.e., judgmental comments) for sexual activity was possibly a manifestation of internalized shame or stigma.

### Theme 03: Barriers to health and social services

Experiencing barriers to health services, especially being dismissed by health and social care practitioners, was an overarching theme across all women-identifying sub-groups. Each sub-group felt these experiences differently based on compounded layers of identity such as English language competency, time spent in Canada, sexual orientation, and/or gender identity. Participants shared both (a) real barriers they had experienced in terms of health access and (b) perceived fears driven by stigma that acted as impediments and barriers to their healthcare.

Cis heterosexual and queer-identifying women shared notable instances of being judged or dismissed by non-MENA doctors in varying healthcare settings as exemplified in this comment. *“I have had a walk-in clinic doctor make me feel guilty for certain behaviors such as not wearing a condom or sleeping with a partner etc*. *This made me feel as though I do not want to disclose as much information with them and from then on I began lying about certain details when discussing sexual health with them*. *Despite this unpleasant treatment I would still make sure to get the medical care I initially wanted (such as STI tests)*.*”* While some women negotiated how they would navigate services to make the most out of the situation, others experienced serious service interruptions and access issues. One participant worrying said, *“I’ve changed doctors like three times in a year because they just didn’t get it*, *or you know*, *didn’t ask the right questions or didn’t want to trust my opinion*.*”*

*Some* participants attributed the dismissal of their health concerns to the age gap between themselves and their healthcare providers. One participant said, *“If you’re talking about anything*, *a lot of these doctors will be very dismissive of what you have to say*, *or what you have to think about the topic*. *And age does play a big factor*, *as well as ethnicity*.*”* Another frustratingly commented, *“Imagine someone that is 60 years old*, *or whatever*, *an older gentleman*, *and I am like 20*, *sitting there*, *like you know explain to me how this is going to go all the way inside*, *how’s this going to protect me*, *what are the things that I need to do*, *what are the things I need to know*?.*”*

The queer-identifying female participants also talked about the need for healthcare providers to be more conscious of sexuality and the particular health risks faced by queer-identifying women. They referenced not being provided with comprehensive information once they discussed their sexuality—which placed them in a low-risk population for STIs and HIV. This group also talked about being dismissed by healthcare workers or not having their health concerns taken seriously as exemplified in this comment:

*I think doctors should normalize talking about sexuality, and not just saying like ‘oh if you’re active try this’. I feel like it should be more normalized*, *as in putting posters around the clinic or just bringing awareness and making the person feel more comfortable opening up about it [sexuality]. Just creating a safe space so you can be open about your cultural identity and your sexual identity.*

Many participants expressed that they would be more comfortable with female physicians. One said, *“One thing that comes to mind to me personally*, *is having a female health care provider often makes me feel more comfortable and makes me feel so I’m more likely to seek these specific services*.*”* Participants also feared being outed to their communities, especially if seeing a doctor who was known within or represented the MENA community. As one participant recalled, *“I ended up changing my family doctor because he wasn’t even Arab*, *but I knew he was Muslim and I was scared he was gonna judge me*. *He was going to think whether I’m like a virgin or not and maybe tell my mom*.*”*

When asked about how comfortable they feel about seeking services from a physician from the MENA community, participants’ responses were mixed. While many shared perceived fears of confidentiality and judgement, others welcomed the opportunity to talk openly with someone from their community, especially around the context of language. One participant clearly stated that *“it is important for me*, *in a sense*, *that I want them to be from the community*, *even though there’s a risk that someone else could find out but I guess for me*, *If I’m talking about something and I’m saying it in Arabic and he understands*, *it makes me feel more comfortable*.*”*

Regardless of ethnicity, the stigma surrounding access to sexual health services in general greatly impacted women’s health service access. Their perceptions and fears also considerably affected how they sought healthcare. As one participant admitted, *“I’m more likely to seek services when I’m not in the same city as my family members*, *so it’s also just like the fear of people finding out has been like a barrier*.*”* Healthcare experiences within the trans women’s group were particularly compounded by transphobia, racism, and English-language barriers as the vast majority identified as newcomers to Canada. Many participants cited experiences where healthcare practitioners tendered discriminatory services as exemplified in this powerful statement, *“I have experienced discrimination in the emergency room*. *I have been called ’it’ and told to stop transitioning and taking hormones*. *My pronouns were not used correctly as well*.*”*

They also discussed their comfort, or lack thereof, in seeing health workers who represented the MENA community. One participant stated, “*imagine that you are going to an Arab doctor*, *automatically you feel that it is better not to talk*. *The psychological factor is very important in such things*.*”* Some cited personal negative experiences. As an example, *“I faced a lot of difficulties because of an Arab doctor who used to treat me horribly because he rejected the LGBT+ community”*

Conversely, like the cisgender women’s group, some participants expressed comfort with and sought health workers from the MENA community. One participant advised that “*we cannot generalize all people or doctors that they are not good*. *My psychiatrist is Egyptian*. *He is the best doctor I have seen*. *He treats me as trans*. *So we cannot generalize*.*”* The trans women participants identified the need for appropriate and accessible mental health services in addition to general health or sexual health related services.

Participants also referenced negative experiences with language interpreters on whom they were relying to convey accurate information about their health. One participant recalled that *“the interpreter was Arab and she would tell her [the doctor] that most [trans] Arabs come here and lie to the government to get benefits*.*”* Some talked about how these experiences perpetuated notions of shame.

### Theme 04: Transphobia and homophobia, specific to the cis LBQ and trans women’s groups

A significant theme across the trans women and cis LBQ women’s groups was their experiences of transphobia and homophobia. These included experiences in the familial, community, and structural systems, as well as through internalized associations of stigma and exclusion. Trans women group participants referenced an isolating lack of support and understanding both within and outside of their cultural community. One participant lamented, “*even at work*, *even everywhere*. *We cannot be how we want to be*, *how we dream to be*. *To live how we want to*, *with independence*. *To say that “I am trans*, *I am gay*.*” We have no fault in it*, *yet the whole population is set on us and wants to judge us*.”

These experiences were not specific to any one institution or system, but crossed the gamut of healthcare, education, and community. Given that many of the women were newcomers, their experiences of discrimination extended to settlement services as well. One participant recalled, “*for organizations that have settlement counsellors*, *if you open a file with them and they find out that you are from the MENA LGBTQ+ community*, *they start to treat you in an unfriendly manner*. *They won’t give you appointments*, *and they will ignore you and cause a lot of issues*.”

Participants frequently compared services and treatment they had experienced in their home countries versus what they expected or felt in Canada. In some instances, they referenced greater comfort in their home countries and spoke of deeply rooted exclusion in Canada. In other instances, participants shared how it was all the same as in “I was restricted with where I was in the Middle East and when I came here, I am still restricted. Nothing changed.” Many voiced their simple desire to be treated with respect and dignity. One woman beseeched organizations “to treat people with the utmost respect. That’s it, that’s all I want. It’s simple. It’s not a lot to ask for from a worker or an organization.”

Cis LBQ women’s group their experiences of homophobia were significant albeit less layered by other barriers such as language. They also demonstrated more ease in navigating the systems around them. A key issue that surfaced for this group was the notion of internalized shame perpetuated by generations of cultural stigma as exemplified in this statement:

*As you’re being raised up you constantly hear your parents, or extended family talking and it’s so ingrained in you and it’s one of those things that you try to unlearn*. *For example, like when you they’re talking about homophobic things there’s so much internalized homophobia, which I’ve worked on getting rid of but there’s massive judgment and stigma associated with it.*

### Theme 05: Self-acceptance and development of personal and peer support networks

Despite the stigma, shame and exclusion felt by a significant number of the women- identifying participants, many also spoke of resiliency and their journey to a place of self- acceptance. They demonstrated a strong awareness of their social space and support networks, and spoke very confidently about their peer groups. The conversations surrounding self-acceptance were often connected to situations in which women found avenues to take control of and/or gain ownership in their identities and relationships (sexual or otherwise). As one trans woman poignantly said,

*When I first came here, I used to be very scared and I couldn’t tell everyone what I am. When I went to school and met people, I felt that I am changing. I was no longer hiding. I started wearing the clothes that make me comfortable and I started telling people that I am trans. If you would like to be my friend, you can be. If you don’t, that’s your problem. I am feeling more confident and I have more confidence in myself despite the racism around me. I don’t care in the end. I got to the point where I don’t care and I feel that I am not wrong within myself. That’s it.*

Layered onto feelings of self-acceptance in relation to gender identity or sexual orientation was a sense of coming to terms with other forms of exclusion women faced such as racism. This was especially true for the cisgender, queer-identifying women whose expressions of self-acceptance extended to them dismantling feelings of internalized shame related to sexuality and their Arab cultural identity. One participant explained it thus: *“How I feel about my cultural identity and being queer is obviously*, *it creates a lot of like internalized homophobia*, *at first*, *if I saw an Arab I was like no way I’m going to interact*, *because they’ll judge*, *but then I met a lot of people who are also like me and that helped*. *I actually embrace it a lot*, *now I like being Arab at the same time*, *because I know a lot of people who are also Arab and queer and we share experiences*.*”* Within the same group, women also talked about having a close community connection with chosen friends and family who either identified with the MENA region or were people of color.

## Discussion

A key objective of the YSMENA study (especially that reported herein) was to understand the intersecting factors that influence and impact the sexual health choices of diaspora MENA youth in Ontario, Canada. Our study yielded valuable insights towards this goal, filled gaps in existing literature, and aided in building an evidence base to inform health programming and policy. Following accepted reporting protocol for qualitative findings, new confirmatory research is introduced in the discussion section to help interpret the data [44].

The five identified themes (see Fig 2) speak to the multiple ways in which MENA youths’ social environments and relationships, in the contexts of their homes, schools, and communities, shaped their identities and influenced their sexual health. To begin, under theme 01, the role of family and mothers on sexual relationships correlates with research in Arab adolescent American communities where the quality of the mother-adolescent relationship was a crucial predictor of adolescent health behavior, and other behavioral problems [18, 45]. Other studies have similarly reported that immigrant Arab mothers were a key determinant of adolescent adjustment, in terms of both health risk and protection [46, 47].

While most of our participants did not talk about marriage, or life partners, their parents’ approval or disapproval figured strongly in their decisions of when and how often they engaged in sexual relationships, if at all. This finding aligns with previous research, which reported that immigrant parents often took an active role in steering their children’s relationships and marriage options [48, 49]. Our participants’ discomfort in talking about sexual health with, or seeking sex and sexuality advice from their parents or other family members is common. Similar experiences were found among racialized youth, particularly the South Asian diaspora community in Toronto whose discomfort and resistance to conversations of sexual health is well documented [49, 50]. Research on family sex communication among Arab American youth has also indicated that silence on topics of sex and sexuality was common and reinforced by social norms thus making peers and friends a more popular source of sex information than family members [51].

The cisgender heterosexual and queer- identifying women in our study talked about the stress of straddling Canadian mainstream spaced (e.g., school and work) while needing to maintain a heightened cultural identity in home spaces. Similar results were reported for Arab youth in Montreal [52]. Between these worlds that they occupied (i.e., their home and the mainstream), topics of sex, sexuality, and sexual health usually materialized in their mainstream space. In other words, they only talked about sex outside their home.

Similar results were reported for Arab youth in Montreal [52]. Between these worlds that they occupied, topics of sex, sexuality and sexual health often fell in their mainstream space. In other words, participants talked about sex outside their home.

Our identified theme of shame, stigma, and the pressure to maintain sexual innocence and/or virginity (theme 02) is well-documented in MENA and Arab communities [17, 48, 53]. Notions of shame profoundly affected individual sexual decision-making and relationships in our study and elsewhere [54]. Other researchers [55] have found that shame and stigma acted as significant instruments for homophobia and transphobia as people with diverse sexual and gender identities were shamed into conforming to heteronormative ways of being. The pressure on women to maintain sexual innocence and adopt ideas of purity were common tools of control in both patriarchal and colonial systems [53, 56]. “Religious conversion, the imposition of rigid gender binaries on colonized peoples, and the criminalization of sexual and gender diversity were strategies of colonial oppression….[Trans women were] educated and taught under a misogynistic, macho, patriarchal scheme of what it means to eb a man or a woman” [57, para. 6]

We found that shame and stigma were a key deterrent to participants’ ability to form and maintain meaningful sexual relationships. Other studies have shown that shaming in sex and sexuality compromise women’s ability to negotiate healthy relationships, leave situations of abuse, seek pleasure out of sexual activity, explore sexuality, and/or comfortably access sexual health resources [58]. Our participants also said that the fear of losing their virginity did not preclude sexual activity, but it did lead to riskier activity. Other research on sexual attitudes and behaviors has similarly reported that young Arab Americans were engaging in a range of sexual activity despite the associated shame and stigma [51]. These findings challenge the assumption that MENA youth do not engage in premarital sexual behaviors.

The stigma associated with sexual health also featured as an important factor in women’s health-seeking behavior as demonstrated in theme 03 related to barriers to health and social services. Fears of being “sexually outed” led some participants to go to great lengths to find services far from their family’s area of residence or to frequently change physicians. This was especially evident for the cisgender heterosexual women who said that their fear of being seen as sexually active or promiscuous strongly determined how and where they accessed sexual health resources The lack of data relating to cisgender heterosexual women from the MENA diaspora in Canada is stark.

Racialized women routinely have their health care concerns dismissed [59, 60]. This correlates with our participants’ experiences. They said being dismissed or undermined by healthcare practitioners and physicians was an ongoing challenge across cultures and identities. From both a societal health and an intersectional standpoint, it is critical that deep seated sexism in health systems is confronted by ensuring that health service practitioners are trained to constructively examine their own bias towards racialized youth who identify across the sexual and gender spectrum.

Many trans women participants talked about traveling significant distances to access services at the limited number of LGBTQ+ positive health clinics situated in Toronto’s downtown core. Research has affirmed that the lack of safe LGBTQ+ service spaces is a common struggle for transgender communities seeking health services [61]. Trans women in our study commented on the compounded barriers around language as many of them were not yet comfortable communicating in English. Lack of competency in English is a noted determinant of newcomer wellbeing in Canada [62]. Our finding points to the need for cultural sensitivity training among language interpreters as they sometimes disparaged participants during medical appointments.

Per theme 04, the transphobia that trans women in our study experienced in spaces of service delivery translates in multiple ways such as pervasive sexual health and HIV-related stigma; heteronormative assumptions; and discriminatory treatment by health professionals, which all but exacerbate their exclusion (see also [63]). From an intersectional lens [25], this is a prime example of multiple determinants compounding to exacerbate marginalization and compromise sexual health care and advice.

While the thematic findings revealed a complex set of struggles and oppressions that women across sub-groups experienced in varying degrees, many also discussed reaching a point of self-awareness and self-acceptance (per theme 05). This was especially the case for the cisgender queer-identifying women who emphasized the value of their chosen families and peers in creating a supportive network with which to navigate difficult terrains. The nascent research on sexual identity development among Arab diaspora has indicated that stigma, oppression, intersectionality around race, sexuality and cultural identity and invisibility are stressors [20].

That said, a significant gap exists in mapping the resiliencies and affirming relationships that they can develop [64]. Queer women’s literature in racialized and/or immigrant diaspora communities has shown that it is not uncommon for strong cultural queer networks to develop as a by-product of limited acceptance in heteronormative cultural community spaces or mainstream or White- dominant queer community spaces [65]. Strongly aligned with these findings, our participants similarly explored alternative ways of conceptualizing community and collectivity across disparate geographic locations. Other researchers are encouraged to further map resilience strategies for this health population. Intersectionality theory will aid this endeavor [25].

To wrap up, our research begins to fill the gap in existing research relating to the sexual health of diaspora MENA youth in Ontario. The Canadian research canon is scathingly sparse with regards to this community. In particular, the literature on racialized newcomer trans women in Ontario is unacceptably low given that this is a priority population for HIV risk that faces major health disparities as compared to other youth sub-populations [63, 66]. Our study is an essential stating point toward building an evidence base to inform future programming.

The latter should focus on the five themes we identified (see Fig 2) upon analyzing qualitative focus group contributions from a unique health population. Health care service providers are urged to become fully cognizant of the extraordinary complicated sexual health life context of cis-and trans-identifying MENA women seeking sexual health care and advice: (a) shame and stigma, (b) fear of being outed, (c) keen desire for respect and retention of dignity during care and (d) deep seated need to minimize prejudicial and discriminatory treatment arising from transphobia and ignorance.

Previous research has considered sexual health, STIs and HIV risk among cisgender university-age students in Egypt [11], Lebanon [12], Morocco [13], Iran [14], and Palestine [67]; however, the geographic specificity of these studies offers little comparative scope for diaspora youth in Ontario. This scenario is exacerbated by the stark lack of data relating to cisgender heterosexual women from the MENA diaspora in Canada. Future Canadian researchers are encouraged to build off our community-based research approach augmented with specially trained PRAs. Researchers are further advised to draw on both intersectional feminism theory and queer theory both of which provided valuable insights into this phenomenon.

Findings from our study contribute to addressing the research lacuna around the cis-and trans-identifying MENA women diaspora in Canada. We offer a much-needed narrative and contextual data that explore the key determinants of MENA youth sexual health. Our findings affirmed research in some areas (e.g., role of families and mothers, shame and stigma, and barriers to health services) and flagged gaps of identified new threads in other areas (e.g., damage of pervasive transphobia, lack of mapping of resiliency strategies, and unexpected increased self-acceptance and sexual identity among Arab diaspora.

That said, additional research is required to triangulate and bolster our findings and build on this work. The intersecting nature of trans women’s health determinants and the transnational nature of their newcomer experiences, with anchored experiences in home countries, may warrant future researchers using a transnational feminist theoretical framework. It draws from postcolonial feminist theories, which emphasize how colonialist legacies have and continue to shape people’s economic, social, and political oppression across the globe [68].

### Study limitations

COVID-19 caused study delays, which were accounted for as soon as online data collection was enabled. However, the online environment posed its own limitations and advantages. Although it enabled some youth to participate who might not have been able to otherwise, the richness of in-person discussion was difficult to emulate. Despite similar training, the PRAs’ varied facilitation styles may have influenced the level and depth of discussion within focus groups. The smaller sample frame typical of qualitative research limited quantitative analysis to descriptive statistics which provided context but precluded regression analysis or comparisons between groups.

## Conclusion

Effective health services reflect a thorough understanding of the communities they are built to serve. To this end, it is imperative that research on MENA diaspora youth continue to grow and add to the currently scarce cannon of evidence available to inform a strong health response. Findings affirm that cisgender and trans women experience complex challenges in relation to how they might access or experience services and resources on sexual health. Family, community, cultural norms, and healthcare systems have considerable impact on young women’s choices. Additionally, intersecting oppressions of homophobia, transphobia, Anti-Arab racism, and xenophobia, against the overarching backdrop of shame and stigma play crucial roles in the sexual lives of young MENA cisgender and trans women.

From an intersectionality perspective, understanding these factors enables tailored and effective health responses that are essential to the health and wellbeing of this growing community. As the number of newcomers to Ontario and Canada from the MENA region increases, their health risks and protective factors will be even more vital. Community-based research that continues our work and explores community experiences against queer and feminist theories, which challenge our conventional understanding of gender, sex and sexuality, will be critical to future knowledge generation and attendant changes to sexual health offerings, access, interventions and healthy policy development.
